# The effect of repeated testing on judgement bias in domestic dogs (*Canis familiaris*)

**DOI:** 10.1007/s10071-022-01689-3

**Published:** 2022-09-12

**Authors:** Clara Wilson, Nathan Hall, Edgar O. Aviles-Rosa, Kerry Campbell, Gareth Arnott, Catherine Reeve

**Affiliations:** 1 Animal Behaviour Centre, School of Psychology, Queen’s University Belfast, Belfast, BT7 1NN UK; 2 Canine Olfaction Research and Education Laboratory, Department of Animal and Food Sciences, Texas Tech University, Box 42141, Lubbock, TX 79409 USA; 3 School of Biological Sciences, Queen’s University Belfast, Belfast, BT7 1NN UK

**Keywords:** Canine, Cognition, Affective state, Judgement bias, Optimism, Pessimism

## Abstract

Judgement bias paradigms are increasingly being used as a measure of affective state in dogs. Approach to an ambiguous stimulus is commonly used as a measure of affect, however, this may also be influenced by learning. This study directly measured the impact of learning on a commonly used judgement bias paradigm in the absence of an affective state manipulation. Dogs (*N* = 15) were tested on a judgement bias task across five sessions. The dogs’ latency to approach a bowl placed in one of three ambiguous locations between non-baited (negative) and baited (positive) locations was measured. Results show that session number had a significant effect on the dogs’ latencies to reach the ambiguous bowl locations, with post-hoc tests revealing that dogs were significantly slower to approach the locations as the number of sessions increased. Session number also had a significant effect on the number of times the dogs did not approach the bowl within 30 s of being released, with the number of no approaches generally increasing across sessions. When dog identity was included as a fixed effect, a significant effect on latency to approach was found, suggesting that some dogs were consistently faster than others across sessions. To assess whether the paradigm produced repeatable results, Intraclass Correlation Coefficients were used. A low degree of reliability was found between latencies to approach each bowl position across sessions. This study demonstrates that dogs learned that the ambiguous locations were not rewarded with repeated exposures, and that this impacted their responses. We conclude that this judgement bias paradigm may require further consideration if applied across multiple exposures and that repeated results should be interpreted with caution as they are likely impacted by learning.

**Supplementary Information:**

The online version contains supplementary material available at 10.1007/s10071-022-01689-3.

## Introduction

Accurately assessing affect in non-human animals is important to answer questions relating to welfare, effect of enrichment, behavioural interventions and psychoactive drug development. A technique that has gained popularity over the last decade is to measure judgement biases in decision making under ambiguity (Harding et al. 2004; Mendl et al. 2009). This method is based on the premise that individuals experiencing different affective states will judge ambiguous stimuli differently. For example, those individuals who are in negatively valanced states are thought to make more negative (“pessimistic”) judgements about ambiguous stimuli than those individuals in more positive (“optimistic”) states (Paul et al. 2005). Since its development, judgement bias tasks have been considered the “gold standard” for measuring affect in non-human animals (Bateson and Nettle 2015). In the last decade, adaptations of the judgement bias protocol have been applied to numerous species, including, but not limited to, sheep (Doyle et al. 2010), chickens (Zidar et al. 2018), horses (Henry et al. 2016), bottlenose dolphins (Clegg et al. 2017), and honeybees (Bateson et al. 2011). In 2010, Mendl et al. adapted the paradigm for application to domestic dogs, whereby a go/no-go task is used and the dog’s latency to approach a food bowl placed in an ambiguous location is measured. The general procedure for this spatial judgement task is as follows: a positive location is paired with food (baited) and a negative location is paired with no reward (non-baited). The most common presentation used with domestic dogs utilises the placement of dog food bowls (e.g., Duranton and Horowitz 2019; Gruen et al. 2019; Kis et al. 2015; Mendl et al. 2010; Müller et al. 2012; Wells et al. 2017. However, see Burman et al. 2011 for a paradigm using coloured card, and Burani et al. 2022 using discrete corridors). In this popular paradigm, the dog is repeatedly shown a dog food bowl in a specific location which either always contains food (e.g., far right-hand side of an otherwise empty room) or is always empty (e.g., far left-hand side). These two presentations are repeated a minimum of fifteen times, with the latency at which the dog approaches the bowl measured (Mendl et al. 2010). During this acquisition phase, it is expected that a learned association will develop, whereby the dog approaches the food bowl on the far right (baited) with a lower latency (i.e., at a faster rate) than the bowl on the far left (non-baited). In the test phase, the dog is presented with ambiguous stimuli over a series of trials, in this case, a bowl in one of three positions between the far right ‘Positive’ and far-left ‘Negative’ positions; either ‘Near Negative’ (NN), ‘Middle’ (M) or ‘Near Positive’ (NP). The dog’s latency to approach these ambiguous stimuli has been widely used as a measure of that dog’s affective state (e.g., Burani et al. 2020; Burman et al. 2011; Gruen et al. 2019; Karagiannis et al. 2015; Mendl et al. 2010; Müller et al. 2012; Wells et al. 2017 and Willen et al. 2019 who used the five bowl protocol as described above, and Duranton and Horowitz 2019; Kis et al. 2015 and Harvey et al. 2020 who used a modified protocol with M as the only ambiguous location). If the dog is faster to approach the ambiguous bowl, it indicates a positive judgement bias (more “optimistic”), whereas a slower approach suggests a negative judgement bias (more “pessimistic”). This protocol has been used successfully in previous studies focussing on domestic dogs to investigate the effects of a range of affect manipulations, including different types of enrichment (Duranton and Horowitz 2019), behavioural, and pharmacological interventions (Karagiannis et al. 2015; Casey et al. 2021). However, studies within the canine literature investigating the reliability and repeatability of this measure are lacking. A potential problem arises when latency is used as a direct measure to interpret affective state, as this may be confounded with learning that may take place across exposures. Assessing the impact of learning is critical to establish the validity of results.

For many types of judgement bias paradigm, learning may be a potential confound as ambiguous stimuli lose their ambiguity across exposures (Burman 2014; Roelofs et al. 2016). Importantly, the ambiguous cues in the canine-adapted judgement bias protocol are not rewarded (they are unbaited, like the Negative bowl location). To mediate the effects of learning, each ambiguous stimulus is presented to the dog only three times per session. However, it is possible that once exposed to the ambiguous stimuli, and receiving no reward, the dog quickly learns that these locations are not rewarding to approach. This may be seen by the dog approaching the bowls with greater latencies (more slowly), or not approaching at all. Within the canine literature, Burani et al. (2020) tested for affect differences between shelter and pet dog populations, and found a learning effect within a single testing session for the ambiguous Middle position on the second presentation, and for the Near Negative position on the third presentation (approaches to the Near Positive location were non-significantly different). These results indicate that dogs may have been learning within one testing session that the ambiguous bowls were not rewarded. However, how these potential learning effects may extend across multiple test exposures has not previously been tested directly.

When considering the impact of learning across exposures, an area of potential interest is whether individual dogs show consistency within the group being tested. For example, dogs may learn over time that the ambiguous bowls are not rewarded and modify their response accordingly, but individual dogs may consistently approach the bowls at a faster or slower rate relative to the group. Barnard et al. (2018) found that certain personality traits were linked to dogs’ performance on the canine-adapted judgement bias task, providing a rationale that some dogs may interpret this task with consistent differences than others. By investigating the role of individual dog within the cohort, we can preliminarily investigate whether the paradigm may be picking up on underlying traits associated with individual dogs.

The focus of this study is assessing the effects of learning on the canine-adapted judgement bias task across multiple sessions. When measuring the impact of an intervention, some studies have utilised repeated measures before and after the intervention, with the judgement bias task being carried out on the same cohort multiple times (e.g., Karagiannis et al. 2015; Duranton and Horowitz 2019). Karagiannis et al. (2015) included a control group in their study looking at the effectiveness of a behavioural and fluoxetine intervention on dogs’ behaviour using a repeated judgement bias task as a measure of affect. It was found that the control group (where no intervention was administered) became slower to respond to the ambiguous bowl locations over time, which is concerning as it suggests that learning effects across sessions could be impacting results. Doyle et al. (2010) investigated learning effects directly with a cohort of sheep, and found that sheep were less likely to approach the ambiguous locations across repeated sessions. Given that the judgement bias protocol is often used to measure dogs’ affect before and after an intervention, meaning that dogs repeat the task across multiple sessions, it is possible that repeated testing is confounded with the effects of ongoing experimental treatment. Therefore, it is important to independently investigate whether the results of this task are impacted by learning effects in the absence of any intervention. As the paradigm grows in popularity, the use of pre- and post-intervention repeated measures is likely to be employed in future studies. We believe that it is essential to study the effects of learning directly in a repeated measures design so that the potential impact can be considered when implementing this paradigm in future.

Therefore, the aim of the current study was to assess the impact of learning on a commonly used judgement bias paradigm in the absence of an affective state manipulation. The objectives of the study were to determine: (1) whether repeated test session (1, 2, 3, 4, 5) and bowl position (Near Negative, Middle or Near Positive) had an effect on dogs’ latencies to reach the ambiguous bowls, (2) whether repeated test session and bowl position had an effect on whether or not dogs approached the ambiguous bowls, (3) whether individual dog identity had an effect on latencies to approach the ambiguous bowls across sessions, 4) whether the dogs’ latencies to approach the ambiguous bowls were consistent across repeated sessions. Based on previous research in surrounding areas, it is predicted that learning will impact dogs’ responses to the ambiguous bowl locations when using the commonly used canine-adapted judgement bias paradigm across repeated sessions. As dogs are not rewarded for approaching the ambiguous bowls, it is predicted that dogs will become slower to approach, or decide to approach less frequently, as they experience a greater number of test sessions.

## Methods

### Animals

Seventeen dogs were recruited for this study, twelve from Queen’s University Belfast (QUB) and five from Texas Tech University (TTU). The QUB dogs were pets owned by members of the community. Dogs were brought by their owners to the QUB laboratory for each session. Owners were recruited via posters placed around QUB campus, an email advertisement and word of mouth. Dogs recruited at TTU were shelter dogs who temporarily were housed at the University to take part in training and research studies before being adopted out to permanent homes. These dogs were selected on the basis of their food motivation, size (medium) and boldness (e.g., willingness to approach and interact with experimenters). These criteria were put in place for olfactory studies that these dogs additionally took part in while at TTU. All TTU dogs were housed in kennels (4.88 m × 2.43 m) in a climate-controlled room. Half of each kennel was outdoors and dogs freely choose access to indoors or outdoors. Dogs had free access to water in the kennel and were fed twice a day (at 8am and 4 pm)*.* Two dogs (both from QUB) were excluded in Session One (for more details see Results Section), resulting in data from 15 dogs. The 15 dogs included seven females (100% spayed) and eight males (75% neutered) with a minimum age of one year and a maximum age of 11.6 years (Mean = 5.00, SD = 3.12). The cohort (QUB and TTU dogs) included a variety of breeds and breed-mixes (Table 1).

**Table 1:** Dog demographics and number of training trials required to meet criterion

Dog Name	Testing Location	Breed	Sex	Age (years)	Session 1	Session 2	Session 3	Session 4	Session 5
Angus	QUB	Rough Collie	M	4	16	15	15	15	15
Bullseye	TTU	Mixed Breed	M	2	19	15	15	15	15
Buster	TTU	Mixed Breed	M	3	22	15	15	15	15
Charles	TTU	Mixed Breed	M	1	15	25	15	15	15
Dale	TTU	Mixed Breed	M	4	21	16	15	15	15
Fingal	QUB	Mixed Breed	M	2.7	15	15	16	15	15
Harvey	QUB	Mixed Breed	M	1.7	15	15	15	-	-
Luna	QUB	Golden Retriever	F	6.7	15	15	15	15	15
Megan	QUB	Border Collie	F	6.5	15	15	15	15	15
Minnie	QUB	Mixed Breed	F	6	18	28	15	15	15
Misty	QUB	Bedlington Terrier	F	11.6	15	15	15	15	15
Poppy	QUB	Siberian Husky	F	7.1	15	15	15	19	15
Sasha	TTU	Mixed Breed	F	9	20	15	15	15	15
Soot	QUB	Mixed Breed	F	2.5	15	26	20	15	15
Walker	QUB	Mixed Breed	M	9	22	15	15	15	15

### Procedure

**Fig. 1:**
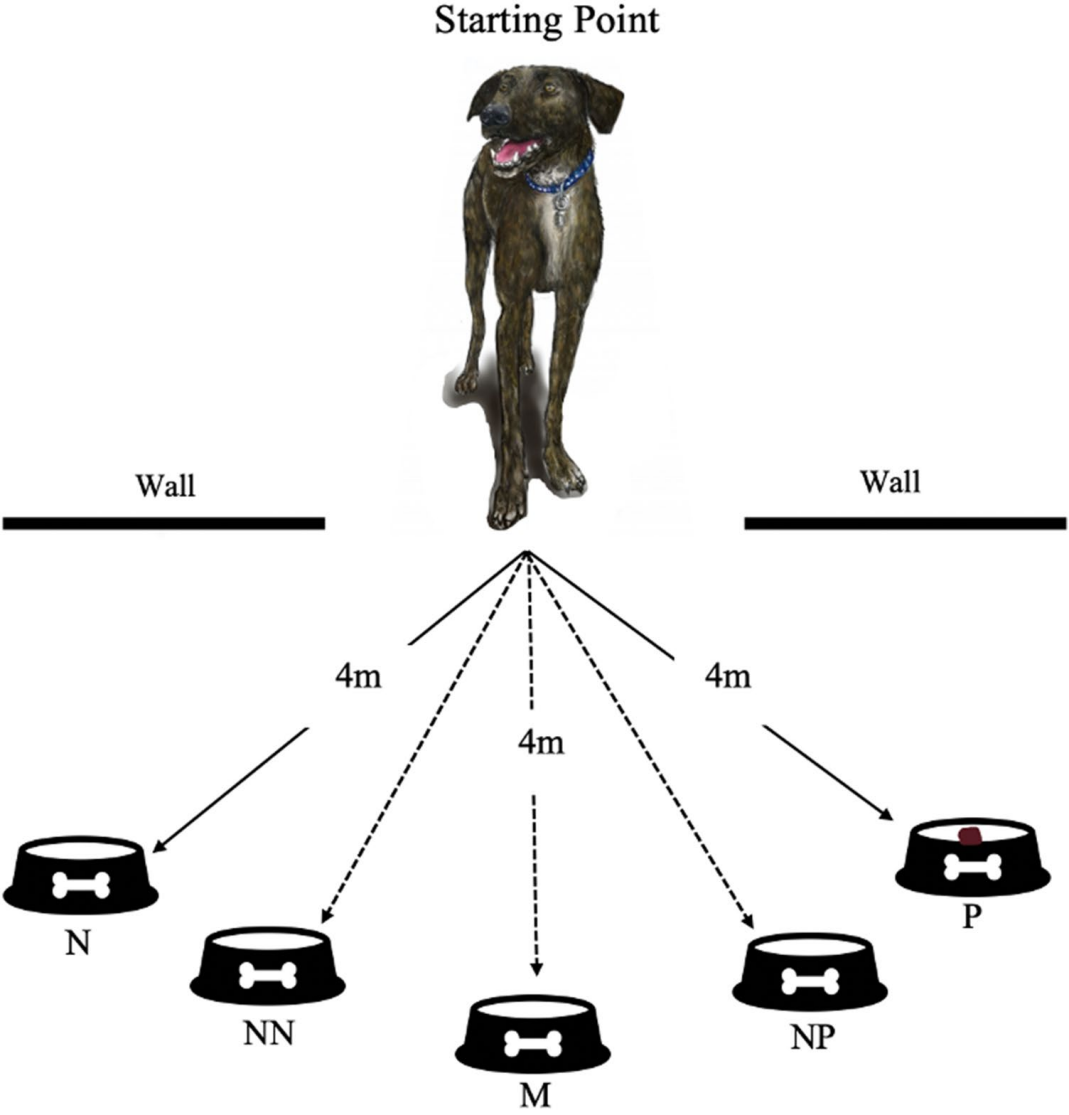
The bowl position layout. N = Negative, NN = Near Negative, M = Middle, NP = Near Positive, P = Positive

The judgement bias procedure was adapted from the protocols described in other studies of canine judgement bias (e.g., Gruen et al. 2019; Mendl et al. 2010; Wells et al. 2017). The start location of the food bowls was marked on the ground using masking tape. A 15 × 15 cm stainless steel dog food bowl was positioned at one of five predetermined locations, four metres from the designated starting position of the dog (Fig. 1). The food bait used was ~ 1 × 1 cm squares of Corned Beef (Princes) for the dogs tested at QUB and ~ 1 × 1 cm rounds of hotdog (Bar S™) for the dogs tested at TTU. Hotdogs were used by TTU after piloting showed that the dogs did not find Corned Beef desirable.

The dog was led to the testing room and positioned in the centre of the room at an equal distance from the predetermined bowl locations (Fig. 1). The dog was released by the researcher (handler) and given the verbal prompt “OK”, allowing the dog to approach the bowl. The latency to reach the bowl was measured via a manual stopwatch. During the acquisition phase, for half of the dogs, the bowl placed in the far-right hand position always contained food (the ‘Positive’ location) and the bowl placed in the far-left location was always empty (the ‘Negative’ location). This location was counterbalanced so that, for the other half of the dogs, these positions were reversed so that the far right was the ‘Negative’ (N) and the far left was the ‘Positive’ (P) location. Whether the Positive bowl was located on the right or left-side of the room was switched between each session to minimise side biases developing. Dogs were initially presented with two consecutive Positive and two consecutive Negative trials. After that, each trial was pseudo-randomly generated to be either Positive or Negative, with no more than two trials of the same type being presented consecutively. The handler led the dog out of the room between trials so as not to see whether food was being placed in the bowl. If the dog did not leave the starting position within three seconds of being released, the handler repeated the prompt “OK”. After the second prompt, if the dog did not approach the bowl within 30 s the trial was recorded as a “no response” and the dog was given a maximum latency score of 30 s.

### Task acquisition

To reach the testing phase, dogs were required to have completed a minimum of 15 acquisition trials and have met the criterion for a learned association between the Positive location and the food reward. The criterion for learned association was that: in the preceding three Positive trials, the dog’s shortest positive latency needed to be shorter than the preceding three Negative trial latencies (Mendl et al. 2010). Within the same session, testing began as soon as the dog reached criterion (the number of trials taken to reach criterion was recorded for each dog—Table 1). If a dog did not reach the criterion within 50 acquisition trials, they did not participate in any testing trials (only one dog was excluded because of this criterion—see Results section).

### Testing

Testing began with two Positive, followed by two Negative, trials. Following that, there was one ambiguous (test probe) trial followed by four Positive or Negative trials (pseudorandom) to maintain and reinforce the associations between Positive and Negative locations. As such, on every fifth trial, a bowl (without a food reward) was placed in one of three ambiguous locations spaced 1.4 m apart along the 4 m arc (Fig. 1). These locations were ‘Near Positive’ (NP) (1.4 m from the Positive location and the Middle), ‘Middle’ (M) (1.4 m from the Near Positive and the Near Negative) and ‘Near Negative’ (NN) (1.4 m from the Middle and Negative positions). Although only one bowl was presented in a single trial, five identical bowls were assigned as either N, NN, M, NP or P. These bowls stayed consistent across dogs and sessions. Nine ambiguous probe test trials were collected within one session in the order: M, NP, NN, NP, NN, M, NN, M, NP (as replicated from Gruen et al. 2019 and Wells et al. 2017).

In line with previous studies, a final trial was carried out as an odour control trial, whereby an empty bowl was placed in the Positive location. This control trial was carried out at the end of each session. We expected the dog to approach the empty bowl with a similar latency as their previous Positive trials. If the dog did not approach at a similar latency to their previous Positive location approaches, it would suggest that they are relying on odour cues to complete the task. Although not carried out in previous studies, an additional control trial was introduced in the current study whereby a baited bowl was positioned in the Negative location. This was done out of curiosity as to whether the dogs’ approach to the Negative bowl would be influenced by the presence of the odour of food, despite having been previously associated with being empty. Here, we expected the dog to show a similar response to the baited Negative bowl as we had seen in previous unbaited Negative trials. If the dog approached the baited Negative bowl at faster rate (e.g., a similar latency to the baited Positive bowl) it would suggest that they were using the presence of olfactory cues to inform their approach decision. This control trial was carried out at the end of each session for the TTU dogs and at the end of Session Five only for the QUB dogs as the QUB data was collected first, and the concept of the baited N trial did not emerge until testing had already started.

The judgement bias protocol was repeated across five sessions. Each session took place seven days apart for the QUB dogs and between five and 19 days apart (Mode = 7 days, Mean = 10.5 days) for the TTU dogs due to differences in study infrastructure.

### Statistical analyses

#### Odour control

A Wilcoxon test for paired samples was carried out on the mean latency to reach the odour control bowl (an unbaited bowl placed in the Positive location) and the mean latency to reach the baited Positive bowl for Sessions One and Five (TTU and QUB dogs included). An additional Wilcoxon test for paired samples was carried out between the mean latency to reach the baited Negative odour control bowl and the mean latency to reach the unbaited Negative bowl for Sessions One and Five (QUB only collected baited Negative data in Session Five, therefore only TTU dogs were analysed for Session One and both cohorts were analysed in Session Five).

#### Trials to reach criterion

To assess the impact of learning in the acquisition phase, a repeated measures t-test was run on the number of training trials it took for the dogs to reach criterion between Session One and Session Two.

#### Bowl differentiation

Prior to investigating the effect of session number on judgement bias task performance, we used a Linear Mixed Model to confirm that the dogs could differentiate between the bowls in the five different locations. Latency data was assessed for normality of distribution. As the data were not normally distributed, the log_10_ transformed data was used. Only the data from the first session was included in this model as the aim of this analysis was just to confirm that dogs were able to discriminate between locations from their first exposure (in line with previous studies). In this model, Latency to Approach the Bowl was the outcome variable, Bowl Position (N, NN, M, NP, P) was a fixed effect and Dog Identity was entered as a random factor. Because Dog Identity was included in the model to account for individual differences in each dog’s relative speed, we used actual latencies rather than an adjusted score. Tests for multiple comparisons (least significant differences) were subsequently run.

#### Effect of repeated session

For all further analyses, only the ambiguous bowl locations (NN, M, NP) were analysed as these were our variables of interest. For all subsequent models, fit was determined using Akaike Information Criterion for small samples (AIC_c_) where smaller scores indicate a better fitting model. Interaction terms were run and dropped from the model on the basis of the AIC_c_ score (for score list per model, see Supplementary Material).

1. Does repeated test sessions and bowl location have an effect on dogs’ latencies to reach the ambiguous bowls?
  To address the first objective, a Linear Mixed Model was used, where Latency to Approach the Bowl was the outcome variable, Session Number (1, 2, 3, 4, 5), Bowl Position (NN, M, NP) and Location of Data Collection (QUB, TT) were the fixed effects and Dog Identity was a random factor. The latency to approach data was assessed for normality of distribution. As the data were not normally distributed, the log_10_ transformed data was used. Tests for multiple comparisons (least significant differences) were subsequently run for statistically significant findings.
2. Does repeated test sessions and bowl location have an effect on whether or not dogs approached the ambiguous bowls?
  To address the second objective, a Generalised Linear Mixed Model with logit link function was used (to account for binary data). Here, Approach Bowl (yes/no) was the outcome variable, Session Number, Bowl Position, Location of Data Collection were fixed effects and Dog Identity was added as a random factor. Tests for multiple comparisons (least significant differences) were subsequently run.
3. Does individual dog identity have an effect on dogs’ latencies to approach the ambiguous bowls across sessions?
  To address the third objective, a Linear Regression Model was used. Here, Latency to Approach the Bowl (transformed using log_10_) was the outcome variable, Dog Identity, Session Number, Location of Data Collected and Bowl Position were all fixed effects. Tests for multiple comparisons (least significant differences) were subsequently run.
4. Are dogs’ latencies to approach the ambiguous bowls consistent across repeated sessions?
  To address the fourth objective, Intraclass Correlation Coefficients (ICC) were used. ICC estimates and their 95% confidence intervals were calculated based on a single measurement, absolute agreement, two-way mixed effects model (McGraw and Wong 1996). This ICC model and type was used because typically only one judgement bias task is used as a measure of canine affect rather than the average of multiple tests (single measurement), the aim of the analysis was test–retest repeatability (absolute agreement), and the selected raters are the only raters of interest (two-way mixed effects model) (McGraw and Wong 1996). Based on the 95% confidence interval of the ICC estimate, values less than 0.5 are considered poor, between 0.5 and 0.75 are moderate, between 0.75 and 0.9 are good, and greater than 0.9 are indicative of excellent reliability (Koo and Li 2016). As it is common to use the judgement bias protocol on only two occasions to test for effects pre- and post an intervention (e.g., Duranton and Horowitz 2019), a further ICC was carried out using Session One and Session Two latencies only, to assess whether approach latencies were consistent across two exposures.

Microsoft SPSS Version 26 and RStudio version 1.4.1103 were used for analyses, with *alpha* = 0.05.

## Results

Of the 17 dogs recruited, two were excluded from analyses. One dog was excluded in Session One for not reaching the testing criterion (they reached 50 acquisition trials without discriminating between P and N). The second dog was excluded in Session One after 39 test trials as they began directing frustration behaviours towards the handler between trials. A third dog completed only three sessions as they had developed repeated marking behaviour in the testing area. Therefore, 15 dogs were included in the analysis: fourteen dogs completed all five sessions and one dog completed three sessions.

Across the five sessions, the number of trials required to reach criterion (with a minimum of 15 trials) varied from 15 to 28 trials (Table 1). Most dogs showed a decrease in number of training trials required from Session One to Session Two, however, three dogs showed an increase in the number of training trials required to meet criterion in from Session One to Session Two. The cohort mean number of trials to reach criterion between Session One (Mean = 17.20, SD = 2.83) and Session Two (Mean = 17.33, SD = 4.70) was non-significant, t(14) = 0.088, *p* = 0.931. From Session Two onwards, most dogs were reaching criterion within the minimum of 15 trials (Table 1).

### Odour control

In Session One, the mean (± SEM) latency (seconds) to reach the odour control bowl (an unbaited bowl placed in the P position) was 2.93 (± 0.19). There was no significant difference between this and the mean latency to reach the baited P bowl, 3.26 (± 0.46), Wilcoxon test for paired samples, *z* =  0.284, *p* = 0.776. In Session Five, the mean (± SEM) latency to reach the unbaited P bowl was 2.48 (± 0.18). There was no significant difference between this and the mean latency to reach the baited P bowl, 2.46 (± 0.15), Wilcoxon test for paired samples, z =  1.468, *p* = 0.142. In Session One, the mean (± SEM) latency to reach the second odour control bowl (a baited bowl placed in the N position) was 30 (± 0.00) (no dog approached the bowl in this trial and therefore all dogs received the maximum latency score of 30). The difference between this and the mean latency to reach the unbaited N bowl, 19.48 (± 3.66), was significant, Wilcoxon test for paired samples, z =  2.023, *p* = 0.043. However, as the dogs were slower to reach the baited N bowl it suggests that they were not relying on odour cues to determine whether or not to approach the bowl. In Session Five, the mean latency to reach the baited N bowl was 24.79 (± 2.78). The difference between this and the mean latency to reach the unbaited N bowl, 20.44 (± 1.93), was not significant, Wilcoxon test for paired samples, z =  1.817, *p* = 0.069. In both sessions the dogs were slower to approach the baited N bowl, which is likely due to the fact that it was the final trial after many unbaited N presentations. The findings from both the unbaited P, and baited N, trials demonstrate that the dogs were not relying on odour cues to determine whether or not to approach the bowl, and that this remained the case across sessions.

### Discrimination between locations

**Fig. 2:**
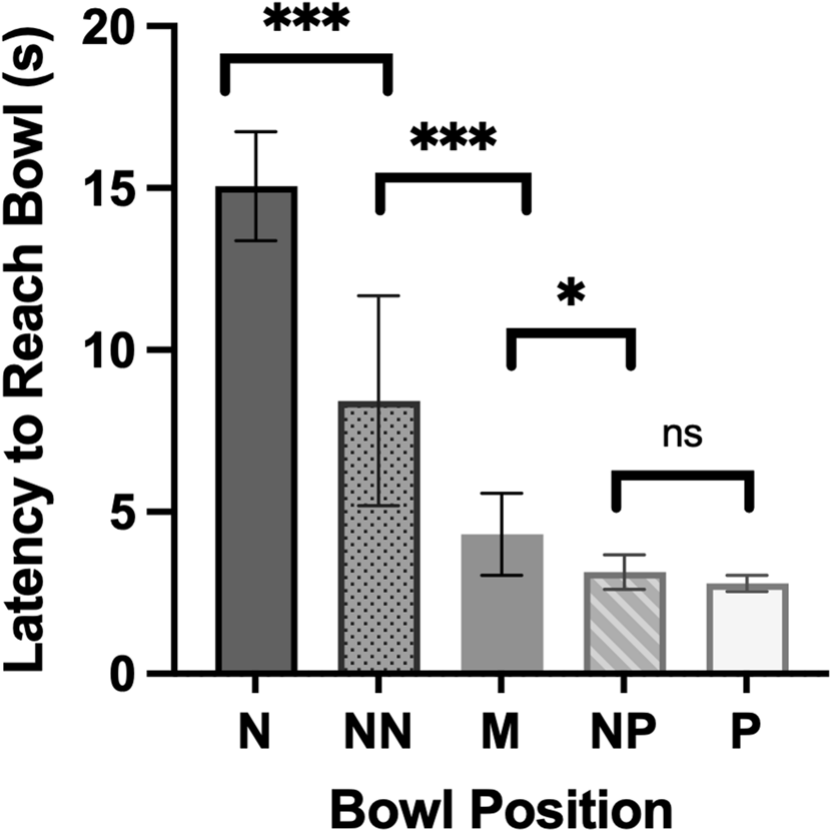
Mean latency (back transformed) to approach each Bowl Position during the first session. Bars represent 95% confidence interval. Tests for multiple comparisons show the significance of differences between positions: “***” indicates *p* < 0.001, “*” indicates *p* < 0.05 and “ns” indicates *p* > 0.05. For all comparisons omitted from figure: *p* < 0.001

A Linear Mixed Model was used to analyse whether dogs were differentiating between the five different bowl locations in Session One. Bowl Position (N, NN, M, NP and P) had a significant effect on the dogs’ latency to reach the bowl (*F*_4,680_ = 277.270, *p* < 0.001). Dogs were fastest to approach the P location, and increased in their latency the further the bowl was positioned from P, suggesting that they were able to discriminate between locations, consistent with the aims of the spatial judgement task (Fig. 2). Test for multiple comparisons using least significant difference showed that the dogs’ latency to approach each Bowl Position was significantly different to the other at the *p* < 0.001 level, with the exception of Middle and Near Positive which was significant at the *p* < 0.05 level (*p* = 0.020) and Near Positive and Positive, which were not significantly different (*p* = 0.258) (see Supplementary Material for full list) (Fig. 2).

### Model 1: Does session number and bowl position have a significant effect on latency to approach the bowl?

**Fig. 3:**
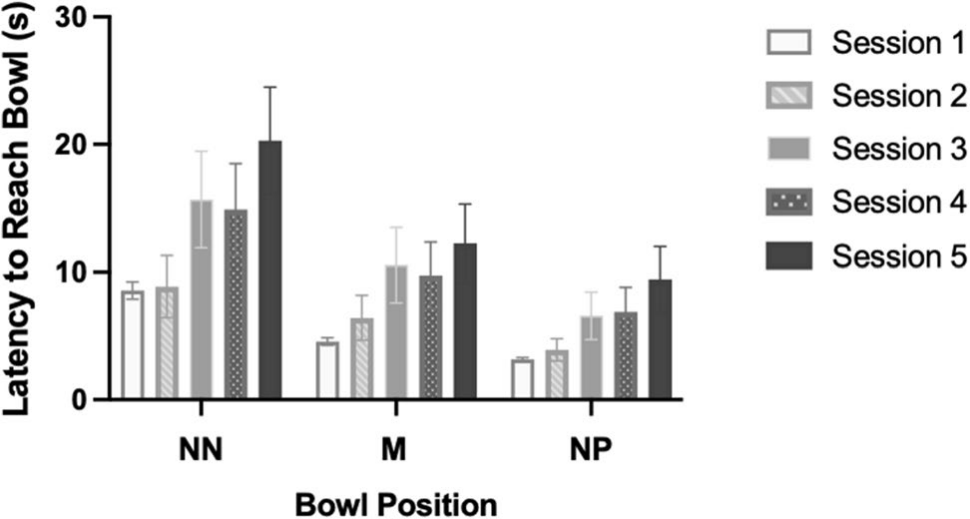
The cohort mean latency (back transformed) to approach each Bowl Position across sessions. Bars represent 95% confidence interval

After reducing variables on the basis of AIC_c_ values, the final model included Session Number, Bowl Position and Location of Data Collection as fixed effects, Dog Identity as a random factor, and Session number by Location of Data Collection as an interaction term. Session Number and Bowl Position were both significantly influencing the dogs’ latencies to approach (*F*_4,645_ = 18.945, *p* < 0.001 and *F*_2,645_ = 48.038, *p* < 0.001, respectively). As a main effect, Location of Data Collection (QUB or TT) was not significant (*F*_1,645_ = 2.049, *p* = 0.153). However, the interaction between Session Number and Location of Data Collection was significant (*F*_4,645_ = 3.637, *p* = 0.006), likely due to the cohort mean latency of the QUB dogs becoming increasingly slower as the session number increased, whereas the cohort mean latency of the dogs tested at TTU showed a small decrease in latency in Sessions Four and Five as compared to Session Three. Tests for multiple comparisons using least significant difference showed that each Session Number pair was significantly different to each other, with the exception of Session One and Session Two (*p* = 0.074), Session Three and Session Four (*p* = 0.458), and Session Three and Session Five (*p* = 0.148) (see Supplementary Material for full list). Each Bowl Position was significantly different to each other at the *p* < 0.001 level (see Supplementary Material for full list). Visualisation of the data shows that, generally, latencies increased across sessions, with the exception of Session Three and Session Four for both the NN and M positions (Fig. 3).

### Model 2: Does session number and bowl position have a significant effect on whether the dog responded?

After reducing variables on the basis of AIC_c_ values, the final model included Session Number and Bowl Position as fixed effects and Dog Identity as a random factor. Session Number and Bowl Position were both significant influences on the dogs’ responses (*F*_4,649_ = 12.477, *p* < 0.001 and *F*_2,649_ = 29.650, *p* < 0.001, respectively) (Table 3). Tests for multiple comparisons using least significant difference showed that each pair of sessions were significantly different in the number of no approaches (*p* < 0.05) with the exception of Session Three and Session Four (*p* = 0.376) and Session Three and Session Five (*p* = 0.108) (see Supplementary Material for full list). When considering Bowl Position, NN and M, and NN and NP, were significantly different in the number of no approaches (*p* < 0.001) whereas M and NP showed a non-significant difference in the number of no approaches (*p* = 0.079) (see Supplementary Material for full list). Visualisation of the data shows that the number of no responses generally increased across sessions (note that one dog was excluded after Session Three, so the total number of bowl presentations is reduced by three for each position in Session Four and Session Five) (Table 3).

**Table 3:** The number of trials where the dog did not approach the bowl within 30 s of being released. Per session, each dog is presented with each ambiguous bowl position three times

	Near Negative	Middle	Near Positive
Session 1 (*n* = 45 per position)	16 (35.5%)	7 (15.6%)	1 (2.2%)
Session 2 (*n* = 45)	18 (40.0%)	12 (26.7%)	6 (13.3%)
Session 3 (*n* = 45)	28 (62.2%)	21 (46.7%)	13 (28.9%)
Session 4 (*n* = 42)	23 (54.8%)	17 (40.5%)	12 (28.6%)
Session 5 (*n* = 42)	31 (73.8%)	20 (47.6%)	17 (40.5%)

### Model 3: Does dog identity have a significant effect on latency to approach bowl?

After reducing variables on the basis of AIC_c_ values, the final model included Dog Identity, Session Number and Bowl Position as fixed effects, and the interaction between Dog Identity and Session Number. It was found that Dog Identity, Session Number and Bowl Position all had significant effects on the dogs’ latency to approach the bowl (Dog Identity: *F*_14,582_ = 5.459, *p* < 0.001, Session Number: *F*_4,582_ = 27.316, *p* < 0.001, Bowl Position: *F*_2,582_ = 51.335, *p* < 0.001). The interaction between Session Number and Dog Identity was significant (*F*_54,582_ = 2.017, *p* < 0.001). This significant interaction term suggests that the effect of session was modified according to Dog Identity, in other words, some dogs differed more across sessions than others.

### Intraclass correlation coefficients

A low degree of reliability was found between latencies to approach each ambiguous bowl position across sessions. When including all five sessions, the degree of reliability was poor (less than 0.5) for the Near Negative position, with the average measure ICC being 0.009 with a 95% confidence interval from − 0.097 to 0.211 (*F*_14,56_ = 1.047). The degree of reliability was poor for the Middle position with the average measure ICC being 0.333 with a 95% confidence interval from 0.148 to 0.576 (*F*_14,56_ = 3.493). The degree of reliability was poor for the Near Positive position with the average measure ICC being 0.241 with a 95% confidence interval from 0.071 to 0.488 (*F*_14,56_ = 2.587).

When comparing only Session One and Session Two latencies, a low degree of reliability was also found. The degree of reliability was poor for the Near Negative position with the average measure ICC being 0.236 with a 95% confidence interval from − 0.211 to 0.601 (*F*_14,14_ = 1.618). The degree of reliability was poor (although approaching moderate) for the Middle position with the average measure ICC being 0.456 with a 95% confidence interval from 0.037 to 0.738 (*F*_14,14_ = 2.679). The degree of reliability was poor for the Near Positive position with the average measure ICC being 0.081 with a 95% confidence interval from − 0.357 to 0.490 (*F*_14,14_ = 1.176) (a table of these results can be found in the Supplementary Material). These results demonstrate that latencies to approach the ambiguous bowl positions largely showed poor consistency, both when considering all five sessions and when considering Session One and Session Two only.

## Discussion

The aim of this study was to determine whether learning had an effect on repeated testing of dogs in a judgement bias task. We used a spatial judgement bias task (the most commonly used protocol), which presented dogs with spatially distinct Positive (baited) and Negative (not baited) stimuli (bowls), and then tested their latency to approach bowls placed in one of three ambiguous locations between Positive and Negative. We found that all but one dog could discriminate between the Positive and Negative locations within 50 training trials, demonstrating that they were able to develop a learned association with the bowl positions as either being baited or unbaited. When looking at the number of trials to reach criterion, three dogs showed an increase in the number of acquisition trials required from Session One to Session Two. This may be due to the fact that the location of the Positive and Negative bowls was switched between each session. It is possible that the dogs had learned which side had been reinforced in the previous session and, therefore, took a greater number of training trials to reach criterion in the second session as they had to reverse this learned bias. However, as a cohort, there was no significant difference in the number of trials taken to reach criterion between Session One and Session Two, and twelve dogs required fewer trials to reach criterion in Session Two. After Session Two, it was found that most dogs required only the minimum number of trials (15) to reach criterion in each session.

The odour control trials (unbaited Positive and baited Negative) showed that the dogs were not using olfactory cues to determine their approach. Dogs were found to have approached the unbaited and baited Positive bowls at non-significantly different latencies, both in Session One and in Session Five. Dogs were found to be significantly slower to approach the baited Negative bowl in Session One, which is likely due to the fact that this was the final trial, carried out after a minimum of 15 acquisition trials and 20 test presentations with an empty bowl in that position. If the odour of food was having an impact on their decision, we would expect to see a sudden decrease in response latency to the baited Negative bowl. However, as we did not observe this, these findings provide strong evidence that the dogs could discriminate between bowl locations and that odour cues did not influence their response.

Our first objective was to investigate whether repeated exposure to the protocol had an impact on the dogs’ latencies to approach the ambiguous bowls. Results from Model One showed that session number had a significant influence on the dogs’ latencies to approach the ambiguous bowl positions. For the dogs tested at Texas Tech University only, we found a slight decrease in latency in Session Four, which resulted in a significant interaction effect of where the data was collected on latency to approach the ambiguous bowl location. This result may have been influenced by a single dog whose responses in Session Four were unexpectedly fast. This finding does, however, raise questions about whether associated differences in the two study locations could influence the level of learning that takes place. Given that we saw a non-significant main effect of location where the data was collected it is believed that these results are likely more so impacted by the individual characteristics of the dogs themselves, rather than the location of testing, as will be discussed below. Figure 3 shows that dogs trended towards becoming slower to approach the ambiguous bowls across sessions. Given that the dogs did not undergo any type of intervention intended to manipulate their affective state, and there were no notable changes to their environment during the study period, this finding likely reflects that dogs were learning that the ambiguous bowl locations were not rewarded. This result supports previous studies that have looked at learning during a judgement bias task. Several studies have reported an effect of learning and cite this as a possible cause of increased latencies in their study subjects (e.g., Sanger et al. 2011; Starling 2012, Starling et al. 2014; Verbeek et al. 2014; for review see Roelofs et al. 2016). It should be considered, however, that the results of Model One also showed that bowl position had a significant effect on the dogs’ responses. As seen in Fig. 3, over the five sessions, the dogs continued to respond to the ambiguous bowls in relation to their proximity to the Positive or Negative locations, for example, the mean latencies to approach the Near Positive bowl were less than the mean latencies to approach the Middle bowl, and the mean latencies to approach the Middle bowl were less than the mean latencies to approach the Near Negative bowl. This finding shows that the dogs were differentiating between the positions across sessions, which demonstrates that the paradigm was, to some degree, working as intended. However, the dogs’ latencies to approach each position generally increased with successive sessions, suggesting that latency to approach is likely not a reliable direct measure of affect across repeated exposures.

The second objective was to examine if repeated testing had an influence on whether or not the dogs chose to approach the ambiguous bowl locations. Model Two was an assessment of the number of no responses, as defined by the dog not approaching the bowl within 30 s. The findings from this model are consistent with the results of Model One, in that the number of no responses generally increased over time, with a slight decrease in no responses seen in Sessions Four, and the highest number of no responses seen in Session Five. Because latency to approach and no responses are related (as a ‘no response’ is given a maximum latency score of 30 s), the decrease found in Session Four is possibly also impacted by a single dog that showed a decrease in no responses and reduced latencies in this session. The number of no responses to the ambiguous bowls were, once again, found to be relative to the bowl's proximity to the Negative or Positive locations, as the number of no responses was highest for bowls in the Near Negative position and lowest for the bowls in the Near Positive position. While the number of no responses generally increased across sessions, the relationship to the Positive and Negative positions was maintained (see Table 2). This suggests that the dogs’ understanding of the Positive and Negative locations were consistent across sessions. It is important to acknowledge that this indicates that the paradigm was functioning as intended, in terms of the dogs’ discrimination of bowl location. However, our findings demonstrate that the increased number of no responses across sessions are a cause for concern if these measures alone are used to measure a dog’s affect. If learning is not taken into consideration, it would appear that these dogs became ‘more pessimistic’ across repeated sessions. Doyle et al. (2010) used a similar protocol whereby sheep would approach feed buckets in one of nine ambiguous locations between a conditioned Positive and Negative bucket. This protocol was repeated across three sessions and it was found that sheep were less likely to approach the feed bucket over time, suggesting that the sheep had learnt that the ambiguous buckets were unrewarded. The results of the current study confirm that such effects are also seen in dogs using this canine-adapted paradigm. Future studies should consider the effect of learning in their design and analyses, or explore methods to counteract learning, to lessen the impact of confounds if using latency or number of approaches as a direct measure of affect.

Model Three looked at Dog Identity as a fixed effect to assess the role of each individual dog’s response to the paradigm. It is possible that the affective state of individual dogs was consistent within the group across sessions. Our results found a significant effect of Dog Identity on latency to approach the ambiguous bowls, in addition to a significant interaction between Dog Identity and Session Number. The significant main effect of Dog Identity suggests that some dogs were consistently faster than others across sessions, which could be contributed to by individual running speed or underlying characteristics specific to each dog. The significant interaction term suggests that individual dog’s responses to the task were moderated by session number. These results could be interpreted as the paradigm detecting some underlying traits specific to each dog, which would be in line with those of Barnard et al. (2018) who found that certain personality traits were linked to dogs’ performance on this canine-adapted judgement bias task. However, to assess a trait (which is stable over time), the protocol must result in highly replicable results (Carter et al. 2013). In this case, the protocol was not able to produce highly replicable results across sessions due to learning (as demonstrated by the Intraclass Correlation Coefficients, discussed below), which may be masking consistency within dogs when taking latencies as a direct measure of affect.

Our final analysis examined the degree of reliability between the dogs’ average latencies to approach the ambiguous bowls across sessions. This was to ascertain if the measure of affect (in this case, latency to approach) was repeatable, or if it changed with each exposure to the task. The Intraclass Correlation Coefficient results showed poor degrees of reliability between sessions, demonstrating that the dogs did not respond consistently. It is important to note that few studies may seek to repeat this protocol as many as five times, as many interventions require only two exposures to the task. When assessing consistency using only Session One and Session Two data, latencies between sessions also showed poor reliability for the NN and NP location, and approaching moderate reliability for the M location. These results suggest that, across even only two exposures, learning is taking place which is impacting latency to approach.

Taken as a whole, these findings suggest that, in studies where dogs are required to repeat a judgement bias task, the results include a learning effect. This learning effect could act as a confound if it interacts with intervention strategies, but, as this study was carried out in the absence of an affect manipulation, the nature of this interaction cannot be defined. It is possible that strong affect manipulation may interact with, and possibly outweigh, these learning effects. For example, Karagiannis et al. (2015) found a positive effect of a fluoxetine and behavioural modification intervention in an experimental group of dogs (they approached the bowls faster over successive sessions post-treatment), whereas the control group became slower with each session. Our finding that dogs become slower to respond, and choose to approach the bowl less, over repeated sessions in the absence of an affect manipulation supports the control group finding of Karagiannis et al. (2015), in addition to raising further questions on what interaction this learning might be having with an experimental treatment. Dogs who show reduced latencies to approach the bowls after a treatment are interpreted to become more “optimistic”, but are also demonstrating less learning of the task than those who undergo no intervention. Future studies may wish to investigate the interaction between affect and learning in more detail.

Several possible solutions to lessen the effects of learning have been previously suggested. One potential method is the use of a secondary reinforcer (e.g., a marker such as a clicker) during training and testing. In this case, approaches to the ambiguous trials would be marked, even though no food reinforcement is given. This technique has been used successfully with bears (Keen et al. 2014) and Rhesus macaques (Bethell et al. 2012) (in this case using a different auditory cue as a secondary reinforcer). Other possible methods include partial reinforcement schedules (e.g., Bateson and Matheson 2007), reducing the number of ambiguous trials (e.g., Duranton and Horowitz, 2019), or rewarding ambiguous trials (for full discussion see Roelofs et al. 2016 and Lagisz et al. 2020). Future studies may wish to investigate methods that could reduce the effects of learning when using the canine-adapted protocol to assess their feasibility when applied to dogs specifically. A method that may warrant further investigation is the use of an “advance key”, where an animal has a third option (in addition to responding, or not responding) which is to choose a “skip to next trial” option. This method has been preliminarily successful at measuring the effects of cognitive enrichment in a shelter dog population (Millar 2013), and may also contribute an ethical improvement beyond having only a positive and negative option. Recently, another novel judgement bias paradigm has been tested with dogs: a five-choice corridor system (Burani et al. 2022). In this paradigm, the ambiguous corridors were presented three times each and were unbaited. However, although not the focus of their study, it was found that the dogs’ latencies to approach the NN and M corridors became significantly slower across the three trial presentations. Burani et al. (2022) conclude that this result can be ascribed to differences in optimism/pessimism rather than learning, therefore further investigation is required. If learning remains a concerning impact in newly developed paradigms then additional amendments to the protocol may be necessary. Overall, results of the current study show that repeated sessions of the currently popular paradigm are impacted by learning effects. If any of the proposed solutions were able to reduce the impacts of learning it may be beneficial that these protocols are integrated into future study designs, especially if researchers plan to utilise repeated testing sessions.

A limitation of the current study is the small number of dogs sampled, which was, in-part, due to data collection taking place amid COVID-19 restrictions which limited dog owner engagement (at Queen’s University Belfast) and access to dogs for re-homing (at Texas Tech University). Future studies may wish to replicate this study with a larger sample size to ensure that the findings are valid. That being said, the found notable increase in latencies and ‘no responses’ across sessions, in addition to the growing body of research highlighting the possible impact of learning effects within judgement bias paradigms, leads us to conclude that repeated unbaited probe trials require additional thought when implemented in repeated measures designs. It should further be acknowledged that the study sample constituted of two groups of dogs who could differ in their affective state or welfare status, as some dogs were pets living in homes and others were being housed in a temporary University shelter. However, the finding that location of data collection (which incorporates all differences between the two groups of dogs) was non-significant as a main effect in the statistical models suggests that this did not have a significant impact on dogs’ approaches. A larger number of dogs in each study group would add greater information, as it is possible that welfare status interacts with ability to learn, as well as affective state. A further potential limitation of this study is that, in carrying out the paradigm in the absence of an affective state manipulation, we have little information on what affective state each dog was in on each day of testing. While the concept of ‘absence of affective state manipulation’ is replicated from Doyle et al. (2010), it could be reasoned that this concept was more achievable in their study species, domestic sheep, who were living in a group housing environment. Given that the dogs included in the current study are housed independently, it is possible that certain dogs had undergone activities or had encounters in the hours prior to testing that may have impacted their affective state, which, in turn, may have impacted their latency to approach in that session. However, because we saw a general increase in latencies to approach the unbaited ambiguous locations across sessions, it is likely that the effects of learning had a significant impact, despite potential differences in individual’s affective state on a session-by-session basis. It is clear that the interaction between affective state and learning has yet to be established fully, and we believe that this emerging area will benefit from future investigation.

## Conclusions

Results show an effect of repeated test exposure on both the dogs’ latencies to approach the ambiguous bowls and the number of no responses. Intraclass Correlation Coefficients between the latencies across sessions showed mostly poor consistency, further suggesting that this canine-adapted judgement bias task does not produce repeatable results across several sessions. Taken as whole, these results indicate that dogs were able to discriminate between bowl positions and interpreted them consistently across sessions in relation to the Positive and Negative locations, however, they learnt that the ambiguous locations are not rewarded which impacted their responses. This finding indicates that changes in judgement bias could be interpreted as differences in learning rather than affect. Future studies may wish to employ methods to counteract such learning effects if using a repeated measure design.

## Supplementary Information

Below is the link to the electronic supplementary material.

Supplementary file1 (DOCX 35 KB)

## Data Availability

The datasets generated during and/or analysed during the current study are available from the corresponding author on reasonable request.
