# Supplementary material for: The effect of repeated testing on judgement bias in domestic dogs (Canis familiaris)
Source: Anim Cogn. 2022 Sep 12;26(2):477–89. doi: 10.1007/s10071-022-01689-3 (PMC9465138; doi:10.1007/s10071-022-01689-3)
Supplement: Supplementary file 1 — Supplementary file1 (DOCX 35 KB) [file 10071_2022_1689_MOESM1_ESM.docx]

**The Effect of Repeated Testing on Judgement Bias in Domestic Dogs (*Canis familiaris*)**

**Clara Wilson^1^, Nathan Hall^2^, Edgar O. Aviles-Rosa^2^, Kerry Campbell^1^, Gareth Arnott^3^, Catherine Reeve^1^**

^1^Animal Behaviour Centre, School of Psychology, Queen’s University Belfast, Belfast, BT7 1NN

^2^Canine Olfaction Research and Education Laboratory, Department of Animal and Food Sciences, Texas Tech University, Box 42141, Lubbock, TX 79409

^3^School of Biological Sciences, Queen’s University Belfast, Belfast, BT7 1NN

Corresponding author:

Clara Wilson, Animal Behaviour Centre, School of Psychology, Queen’s University Belfast, Belfast, BT7 1NN

cwilson75@qub.ac.uk

Table 1. AIC_c_ scores for each model

|  | **AIC_c_ Score** | | |
| --- | --- | --- | --- |
|  | **Model 1** | **Model 2** | **Model 3** |
| All interaction terms | 721.858 | 3257.931 | 747.967 |
| Only significant interaction terms | **697.63** | 3084.668 | **695.357** |
| No interaction terms | 700.029 | 3021.770 | 710.670 |
| Location (non-significant) removed | 698.670 | **3019.366** | 710.670 |

N.B. Bold indicates the best fitting model.

Table 2: Tests for multiple comparisons with least significant differences for each Bowl Position in Session One

| **Bowl Position Pairwise Contrasts** | **Contrast Estimate** | **Standard Error** | **t** | **df** | **Adj. Significance** | **95% Confidence Interval** | |
| --- | --- | --- | --- | --- | --- | --- | --- |
|  |  |  |  |  |  | **Lower Bound** | **Upper Bound** |
| Negative - Near Negative | 0.251 | 0.045 | -5.683 | 680 | < 0.001 | 0.164 | 0.339 |
| Negative - Middle | 0.543 | 0.045 | 12.183 | 680 | < 0.001 | 0.455 | 0.630 |
| Negative - Near Positive | 0.680 | 0.045 | 15.268 | 680 | < 0.001 | 0.164 | 0.339 |
| Negative - Positive | 0.731 | 0.023 | 32.114 | 680 | < 0.001 | 0.686 | 0.775 |
| Near Negative -Negative | -0.251 | 0.045 | -5.638 | 680 | < 0.001 | -0.339 | -0.164 |
| Near Negative -Middle | 0.292 | 0.059 | 4.964 | 680 | < 0.001 | 0.176 | 0.407 |
| Near Negative - Near Positive | 0.429 | 0.059 | 7.304 | 680 | < 0.001 | 0.314 | 0.544 |
| Near Negative - Positive | 0.479 | 0.045 | 10.767 | 680 | < 0.001 | 0.392 | 0.567 |
| Middle - Negative | -0.543 | 0.045 | -12.183 | 680 | < 0.001 | -0.630 | -0.455 |
| Middle - Near Negative | -0.292 | 0.059 | -4.964 | 680 | < 0.001 | -0.407 | -0.176 |
| Middle - Near Positive | 0.137 | 0.059 | 2.340 | 680 | 0.020 | 0.022 | 0.253 |
| Middle - Positive | 0.188 | 0.045 | 4.219 | 680 | < 0.001 | 0.100 | 0.275 |
| Near Positive - Negative | -0.680 | 0.045 | -15.268 | 680 | < 0.001 | -0.768 | -0.593 |
| Near Positive - Near Negative | -0.429 | 0.059 | -7.304 | 680 | < 0.001 | -0.554 | -0.314 |
| Near Positive - Middle | -0.137 | 0.059 | -2.340 | 680 | 0.020 | -0.253 | -0.022 |
| Near Positive - Positive | 0.050 | 0.059 | -7.304 | 680 | 0.258 | -0.037 | 0.138 |
| Positive - Negative | -0.731 | 0.023 | -32.114 | 680 | < 0.001 | -0.775 | -0.686 |
| Positive - Near Negative | -0.479 | 0.045 | -10.767 | 680 | < 0.001 | -0.567 | -0.392 |
| Positive - Middle | -0.188 | 0.045 | -4.219 | 680 | < 0.001 | -0.275 | -0.100 |
| Positive -Near Positive | -0.050 | 0.045 | -1.132 | 680 | 0.258 | -0.138 | 0.037 |

N.B. The least significance adjusted significance level is 0.05.

Table 3: Tests for multiple comparisons with least significant differences for each Session Number in Model One

| **Session Number Pairwise Contrasts** | | **Contrast Estimate** | **Standard Error** | **t** | **df** | **Adj. Significance** | **95% Confidence Interval** | |
| --- | --- | --- | --- | --- | --- | --- | --- | --- |
|  |  |  |  |  |  |  | **Lower Bound** | **Upper Bound** |
| 1 | 2 | -0.091 | 0.051 | -1.790 | 645 | 0.074 | -0.190 | 0.009 |
|  | 3 | -0.304 | 0.051 | -6.008 | 645 | < 0.001 | -0.404 | -0.205 |
|  | 4 | -0.266 | 0.051 | -5.201 | 645 | < 0.001 | -0.367 | -0.166 |
|  | 5 | -0.378 | 0.051 | -7.390 | 645 | < 0.001 | -0.479 | -0.278 |
| 2 | 1 | 0.091 | 0.051 | 1.790 | 645 | 0.074 | -0.009 | 0.190 |
|  | 3 | -0.214 | 0.051 | -4.219 | 645 | < 0.001 | -0.313 | -0.114 |
|  | 4 | -0.038 | 0.051 | -3.431 | 645 | 0.001 | -0.276 | -0.075 |
|  | 5 | -0.288 | 0.051 | -5.620 | 645 | < 0.001 | -0.388 | -0.187 |
| 3 | 1 | 0.304 | 0.051 | 6.008 | 645 | < 0.001 | 0.205 | 0.404 |
|  | 2 | 0.214 | 0.051 | 4.219 | 645 | < 0.001 | 0.114 | 0.313 |
|  | 4 | 0.038 | 0.051 | 0.740 | 645 | 0.459 | -0.063 | 0.138 |
|  | 5 | -0.074 | 0.051 | -1.448 | 645 | 0.148 | -0.175 | 0.026 |
| 4 | 1 | 0.266 | 0.051 | 5.201 | 645 | < 0.001 | 0.166 | 0.367 |
|  | 2 | 0.176 | 0.051 | 3.431 | 645 | 0.001 | 0.075 | 0.276 |
|  | 3 | -0.038 | 0.051 | -0.740 | 645 | 0.459 | -0.138 | 0.063 |
|  | 5 | -0.112 | 0.052 | -2.174 | 645 | 0.030 | -0.213 | -0.011 |
| 5 | 1 | 0.378 | 0.051 | 7.390 | 645 | < 0.001 | 0.278 | 0.479 |
|  | 2 | 0.288 | 0.051 | 5.620 | 645 | < 0.001 | 0.187 | 0.388 |
|  | 3 | 0.074 | 0.051 | 1.448 | 645 | 0.148 | -0.026 | 0.175 |
|  | 4 | 0.112 | 0.052 | 2.174 | 645 | 0.030 | 0.011 | 0.213 |

N.B. The least significance adjusted significance level is 0.05.

| Table 4: Tests for multiple comparisons with least significant differences for each Bowl Position in Model One | | | | | | | |
| --- | --- | --- | --- | --- | --- | --- | --- |
| **Bowl Position Pairwise Contrasts** | **Contrast Estimate** | **Standard Error** | **t** | **df** | **Adj. Significance** | **95% Confidence Interval** | |
|  |  |  |  |  |  | **Lower Bound** | **Upper Bound** |
| Near Negative -Middle | 0.198 | 0.037 | 5.275 | 645 | < 0.001 | 0.124 | 0.271 |
| Near Negative -Near Positive | 0.367 | 0.037 | 9.792 | 645 | < 0.001 | 0.293 | 0.441 |
| Middle - Near Negative | -0.198 | 0.037 | -5.275 | 645 | < 0.001 | -0.271 | -0.124 |
| Middle - Near Positive | 0.169 | 0.037 | 4.517 | 645 | < 0.001 | 0.096 | 0.243 |
| Near Positive - Near Negative | -0.367 | 0.037 | -9.792 | 645 | < 0.001 | -0.441 | -0.293 |
| Near Positive - Middle | -0.169 | 0.037 | -4.517 | 645 | < 0.001 | -0.243 | -0.096 |

N.B. The least significance adjusted significance level is 0.05.

| Table 5: Tests for multiple comparisons with least significant differences for each Session Number in Model Two | | | | | | | | |
| --- | --- | --- | --- | --- | --- | --- | --- | --- |
| **Session Number Pairwise Contrasts** | | **Contrast Estimate** | **Standard Error** | **t** | **df** | **Adj. Significance** | **95% Confidence Interval** | |
|  |  |  |  |  |  |  | **Lower Bound** | **Upper Bound** |
| 1 | 2 | -0.102 | 0.046 | -2.237 | 650 | 0.026 | -0.191 | -0.012 |
|  | 3 | -0.289 | 0.054 | -5.299 | 650 | < 0.001 | -0.396 | -0.182 |
|  | 4 | -0.232 | 0.054 | -4.287 | 650 | < 0.001 | -0.339 | -0.126 |
|  | 5 | -0.395 | 0.057 | -6.884 | 650 | < 0.001 | -0.507 | -0.282 |
| 2 | 1 | 0.102 | 0.046 | 2.237 | 650 | 0.026 | 0.012 | 0.191 |
|  | 3 | -0.187 | 0.058 | -3.196 | 650 | < 0.001 | -0.302 | -0.072 |
|  | 4 | -0.130 | 0.058 | -2.234 | 650 | 0.026 | -0.245 | -0.016 |
|  | 5 | -0.293 | 0.061 | -4.798 | 650 | < 0.001 | -0.413 | -0.173 |
| 3 | 1 | 0.289 | 0.054 | 5.299 | 650 | < 0.001 | 0.182 | 0.396 |
|  | 2 | 0.187 | 0.058 | 3.196 | 650 | < 0.001 | 0.072 | 0.302 |
|  | 4 | 0.057 | 0.064 | 0.885 | 650 | 0.376 | -0.069 | 0.182 |
|  | 5 | -0.106 | 0.066 | -1.610 | 650 | 0.108 | -0.235 | 0.023 |
| 4 | 1 | 0.232 | 0.054 | 4.287 | 650 | < 0.001 | 0.126 | 0.339 |
|  | 2 | 0.130 | 0.058 | 2.234 | 650 | 0.026 | 0.016 | 0.245 |
|  | 3 | -0.057 | 0.064 | -0.885 | 650 | 0.376 | -0.182 | 0.069 |
|  | 5 | -0.163 | 0.066 | -2.476 | 650 | 0.014 | -0.292 | -0.034 |
| 5 | 1 | 0.395 | 0.057 | 6.884 | 650 | < 0.001 | 0.282 | 0.507 |
|  | 2 | 0.293 | 0.061 | 4.798 | 650 | < 0.001 | 0.173 | 0.413 |
|  | 3 | 0.106 | 0.066 | 1.610 | 650 | 0.108 | -0.023 | 0.235 |
|  | 4 | 0.163 | 0.066 | 2.174 | 650 | 0.014 | 0.034 | 0.292 |
| N.B. The least significance adjusted significance level is 0.05. | | | | | | | | |

| Table 6: Tests for multiple comparisons with least significant differences for each Bowl Position in Model Two | | | | | | | |
| --- | --- | --- | --- | --- | --- | --- | --- |
| **Bowl Position Pairwise Contrasts** | **Contrast Estimate** | **Standard Error** | **t** | **df** | **Adj. Significance** | **95% Confidence Interval** | |
|  |  |  |  |  |  | **Lower Bound** | **Upper Bound** |
| Near Negative -Middle | 0.284 | 0.048 | 5.959 | 650 | < 0.001 | 0.190 | 0.377 |
| Near Negative -Near Positive | 0.353 | 0.046 | 7.670 | 650 | < 0.001 | 0.263 | 0.443 |
| Middle -Near Negative | -0.284 | 0.048 | -5.959 | 650 | < 0.001 | -0.377 | -0.190 |
| Middle - Near Positive | 0.069 | 0.039 | 1.757 | 650 | 0.079 | -0.008 | 0.146 |
| Near Positive - Near Negative | -0.353 | 0.046 | -7.670 | 650 | < 0.001 | -0.443 | -0.263 |
| Near Positive - Middle | -0.069 | 0.039 | -1.757 | 650 | 0.079 | -0.146 | 0.008 |

The least significance adjusted significance level is 0.05.

| Table 7. Intraclass Correlation Coefficient results | | | | | | | |
| --- | --- | --- | --- | --- | --- | --- | --- |
| **Bowl Position** | **ICC** | **F** | **df1** | **df2** | ***p* value** | **95% Confidence Interval** | |
|  |  |  |  |  |  | **Lower Bound** | **Upper Bound** |
| M  All Sessions | 0.333 | 3.493 | 14 | 56 | < 0.001 | 0.148 | 0.576 |
| NN  All Sessions | 0.009 | 1.047 | 14 | 56 | 0.423 | -0.097 | 0.211 |
| NP  All Sessions | 0.241 | 2.587 | 14 | 56 | 0.006 | 0.071 | 0.488 |
| M  Sessions 1-2 | 0.456 | 2.679 | 14 | 14 | 0.038 | 0.038 | 0.739 |
| NN  Sessions 1-2 | 0.236 | 1.618 | 14 | 14 | 0.189 | -0.211 | 0.602 |
| NP  Sessions 1-2 | 0.081 | 1.176 | 14 | 14 | 0.383 | -0.357 | 0.490 |
